# Multilevel Hierarchical Kernel Spectral Clustering for Real-Life Large Scale Complex Networks

**DOI:** 10.1371/journal.pone.0099966

**Published:** 2014-06-20

**Authors:** Raghvendra Mall, Rocco Langone, Johan A. K. Suykens

**Affiliations:** ESAT-STADIUS, KU Leuven, Leuven, Belgium; Cinvestav-Merida, Mexico

## Abstract

Kernel spectral clustering corresponds to a weighted kernel principal component analysis problem in a constrained optimization framework. The primal formulation leads to an eigen-decomposition of a centered Laplacian matrix at the dual level. The dual formulation allows to build a model on a representative subgraph of the large scale network in the training phase and the model parameters are estimated in the validation stage. The KSC model has a powerful out-of-sample extension property which allows cluster affiliation for the unseen nodes of the big data network. In this paper we exploit the structure of the projections in the eigenspace during the validation stage to automatically determine a set of increasing distance thresholds. We use these distance thresholds in the test phase to obtain multiple levels of hierarchy for the large scale network. The hierarchical structure in the network is determined in a bottom-up fashion. We empirically showcase that real-world networks have multilevel hierarchical organization which cannot be detected efficiently by several state-of-the-art large scale hierarchical community detection techniques like the Louvain, OSLOM and Infomap methods. We show that a major advantage of our proposed approach is the ability to locate good quality clusters at both the finer and coarser levels of hierarchy using internal cluster quality metrics on 7 real-life networks.

## Introduction

Large scale complex networks are ubiquitous in the modern era. Their presence spans a wide range of domains including social networks, trust networks, biological networks, collaboration networks, financial networks etc. A complex network can be represented as a graph 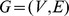 where *V* represent the vertices or nodes and *E* represents the edges or interaction between these nodes in this network. Many real-life complex networks are scale-free [1], follow the power law [2] and exhibit community like structure. By community like structure one means that nodes within one community are densely connected to each other and sparsely connected to nodes outside that community. The large scale network consists of several such communities. This problem of community detection in graphs has received wide attention from several perspectives [3]–[14].

The community structure exhibited by the real world complex networks often have an inherent hierarchical organization. This suggests that there should be multiple levels of hierarchy in these real-life networks with good quality clusters at each level. In other words, there exist meaningful communities at refined as well as coarser levels of granularity in this multilevel hierarchical system of the real-life networks.

A state-of-the-art hierarchical community detection technique for large scale networks is the Louvain method [15]. It uses a popular quality function namely *modularity* (Q) [3], [5], [6], [16] for locating modular structures in the network in a hierarchical fashion. Modularity measures the difference between a given partition of a network and the expectation of the same partition for a random network. By optimizing modularity, they obtain the modular structures in the network. However, it suffers from a drawback namely the resolution limit problem [17]–[19]. The issue of resolution limit arises because the optimization of modularity beyond a certain resolution is unable to identify modules even as distinct as cliques which are completely disconnected from the rest of the network. This is because modularity fixes a global resolution to identify modules which works for some networks but not others.

Recently the authors of [20] show that methods trying to use variants of modularity to overcome the resolution limit problem, still suffer from the resolution limit. They propose an alternative algorithm namely OSLOM [21] to avoid the issue of resolution. However, in our experiments we observe that OSLOM works well for benchmark synthetic networks [4] but in case of real-life networks it is unable to detect quality coarse clusters. We also evaluate another state-of-the-art hierarchical community detection technique called the Infomap method [7]. The Infomap method uses an information theoretic approach to hierarchical community detection. It uses the probability flow of random walks as a substitute for information flow in real-life networks. It then fragments the network into modules by compressing a description of the probability flow.

Spectral clustering methods [10]–[14] belong to the family of unsupervised learning algorithms where clustering information is obtained by the eigen-decomposition of the Laplacian matrix derived from the affinity matrix (*S*) for the given data. A drawback of these methods is the construction of the large affinity matrix for the entire data which limits the feasibility of the approach to small sized data. To overcome this problem, a kernel spectral clustering (KSC) formulation based on weighted kernel principal component analysis (kPCA) in a primal-dual framework was proposed in [22]. The weighted kPCA problem is formulated in the primal in the context of least squares support vector machines [23] which results in eigen-decomposition of a centered Laplacian matrix in the dual. As a result, a clustering model is obtained in the dual. This model is build on a subset of the original data and has a powerful out-of-sample extension property. This property allows cluster affiliation for unseen data.

The KSC method was applied for community detection in graphs by [24]. However, their subset and model selection approach was computationally expensive and memory inefficient. Recently, the KSC method was extended for big data networks in [25]. The method works by building a model on a representative subgraph of the large scale network. This subgraph is obtained by the fast and unique representative subset (FURS) selection technique as proposed in [26]. During the model selection stage, the model parameters are estimated along with determining the number of clusters *k* in the network. A self-tuned KSC model for big data networks was proposed in [27]. The major advantage of the KSC method is that it creates a model which has a powerful out-of-sample extensions property. Using this property, we can infer community affiliation for unseen nodes of the whole network.

In [28], the authors used multiple scales of the kernel parameter *σ* to determine the hierarchies in the data using KSC approach. However, in this approach the clustering model is trained for different values of (*k*,*σ*) and evaluated for the entire dataset using the out-of-sample extension property. Then, a map is created to match the clusters at two levels of hierarchy. As stated by the authors in [28], during a merge there might be some data points of the merging clusters that go into a non-merging cluster which is then forced to join the merging cluster of the majority. In this paper, we overcome this problem and generate a natural hierarchical organization of the large scale network in an agglomerative fashion.

The purpose of hierarchical community detection is to automatically locate multiple levels of granularity in the network with meaningful clusters at each level. The KSC method has been used effectively to obtain flat partitioning in real-world networks [24], [25], [27]. In this paper, we exploit the structure of the eigen-projections derived from the KSC model. The projections of the validation set nodes in the eigenspace is used to create an iterative set of affinity matrices resulting in a set of increasing distance thresholds (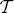). Since the validation set of nodes is a representative subset of the large scale network [26], we use these distance thresholds (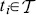) on the projections of the entire network obtained as a result of the out-of-sample extension property of the KSC model. These distance thresholds, when applied in an iterative manner, provide a multilevel hierarchical organization for the entire network in a bottom-up fashion. We show that our proposed approach is able to discover good quality coarse as well as refined clusters for real-life networks.

**Figure 1:**
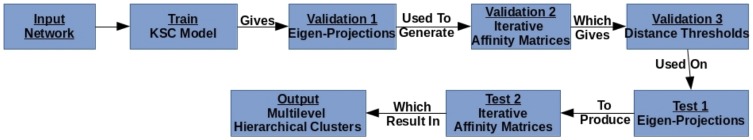
Steps undertaken by the MH-KSC algorithm.

**Figure 2:**
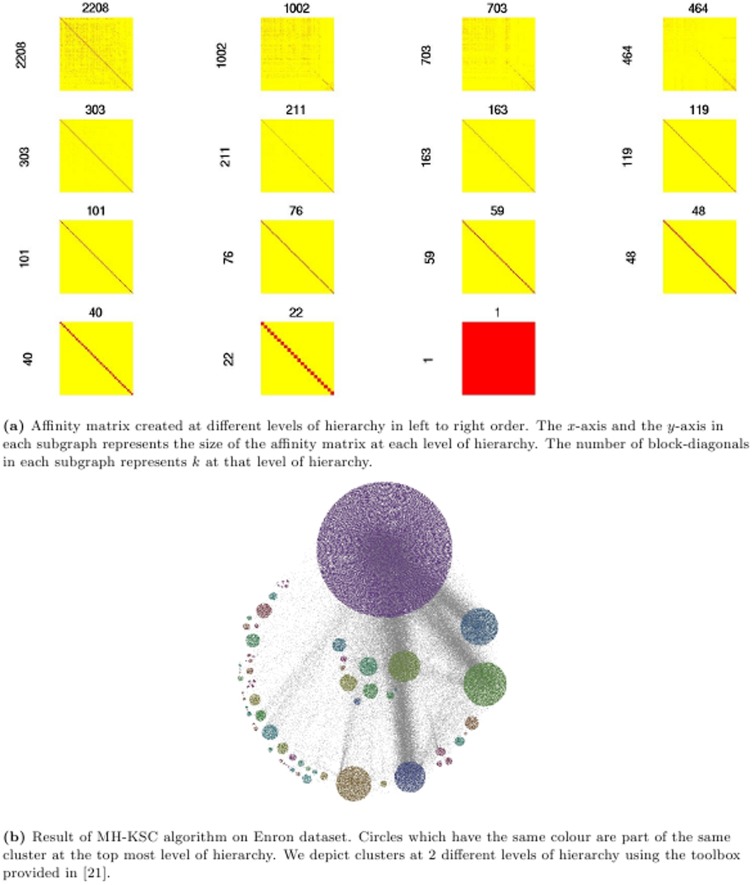
Result of proposed MH-KSC approach on the Enron network.

There are some methods that optimize weighted graph cut objectives [29]–[31] to provide multilevel clustering for the large scale network. However, these methods suffer from the problem of determining the right value of *k* which is user defined. In real-world networks the value of *k* is not known beforehand. So in our experiments, we evaluate the proposed multilevel hierarchical kernel spectral clustering (MH-KSC) algorithm against the Louvain, Infomap and OSLOM methods. These methods automatically determine the number of clusters (*k*) at each level of hierarchy. Figure 1 provides an overview of steps involved in the MH-KSC algorithm and Figure 2 depicts the result of our proposed MH-KSC approach on email network (Enron).

In all our experiments we consider unweighted and undirected networks. All the experiments were performed on a machine with 12 Gb RAM, 2.4 GHz Intel Xeon processor. The maximum size of the kernel matrix that is allowed to be stored in the memory of our PC is 10,000×10,000. Thus, the maximum cardinality of our training and validation sets can be 10,000. We use 15% of the total nodes as size of training and validation set (if less than 10,000) based on experimental findings in [32]. We make use of the procedure provided in [25] to divide the data into chunks in order to extend our proposed approach to large scale networks. There are several steps in the proposed methodology which can be implemented on a distributed environment. We describe this in detail later.

## Kernel Spectral Clustering (KSC) Method

We first summarize the notations used in the paper.

### Notations

1. A graph is mathematically represented as 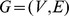 where *V* represents the set of nodes and 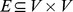 represents the set of edges in the network. Physically, the nodes represent the entities in the network and the edges represent the relationship between these entities.
2. The cardinality of the set *V* is denoted as *N*.
3. The training, validation and test set of nodes is given by 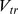, 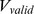 and 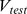 respectively.
4. The cardinality of the training, validation and test set is given 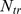, 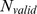, 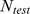.
5. The adjacency list corresponding to each vertex 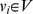 is given by 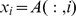.
6. *maxk* is the maximum number of eigenvectors that we want to evaluate.

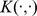
 represents the positive definite kernel function.
8. The matrix *S* represents the affinity or similarity matrix.
9. *P* represents the latent variable matrix containing the eigen-projections.
10. *h* represents the *h^th^* level of hierarchy and *maxh* stands for the coarsest level of hierarchy.
11. Set *C* comprises multilevel hierarchical clustering information.
12. Coarsest level of hierarchy corresponds to fine grained clusters and finer levels of hierarchy correspond to coarse clusters.

### KSC methodology

Given a graph *G*, we use the fast and unique representative subset (FURS) selection [26] technique to obtain training and validation set of nodes 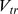 and 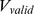. FURS [26] is a deterministic subgraph selection technique where nodes with high degree centrality are greedily selected from most or all the communities in the network. Nodes with high degree centrality are usually located at the center, away from the periphery of the network and can better capture the inherent community structure. Since our goal is a locate multilevel hierarchical clustering in the large scale network, it is essential that the training and validation set are representative of the underlying community structure of the network. A detailed description of the FURS approach and its comparison with other state-of-the-art subset selection techniques is provided in [26].

We use 15% of the total nodes as size of training and validation set (if less than 10,000 otherwise 10,000 nodes) based on experimental findings in [32]. Firstly, we apply FURS to obtain the training set of nodes 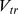. Once these nodes are selected in the training set we remove these nodes from the network but maintain the topology (degree distribution) of the network. We then apply FURS again to obtain the validation set of nodes 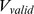. Thus, both these sets 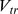 and 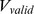 are selected such that they retain the inherent community structure of the large scale network. We then use the entire large scale network as the test set 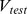.

For 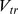 training nodes the dataset is given by 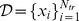, 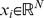. The adjacency list *x_i_* can efficiently be stored into memory as real-world networks are highly sparse and have limited connections for each node 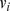.

Given 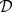 and *maxk*, the primal formulation of the weighted kernel PCA [22] is given by:
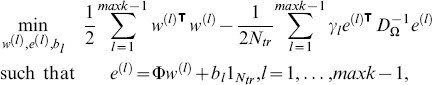
(1)where 
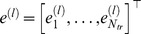
 are the projections onto the eigenspace, 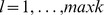-1 indicates the number of score variables required to encode the *maxk* clusters. However, it was shown in [27] that we can discover more than *maxk* communities using these *maxk*-1 score variables. 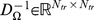 is the inverse of the degree matrix associated to the kernel matrix Ω with 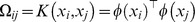. Φ is the 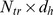 feature matrix such that 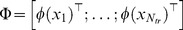 and 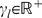 is the regularization constant. We note that 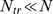 i.e. the number of nodes in the training set is much less than the total number of nodes in the large scale network.

The kernel matrix Ω is constructed by calculating the similarity between the adjacency list of each pair of nodes in the training set. Each element of Ω, defined as 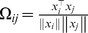 is calculated by estimating the cosine similarity between the adjacency lists 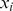 and 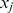 using notions of set intersection and union. This corresponds to using a normalized linear kernel function 
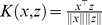
 [23].

The primal clustering model is then represented by:
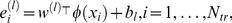
(2)where 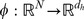 is the feature map i.e. a mapping to high-dimensional feature space *d_h_* and *b_l_* are the bias terms, 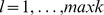-1. For large scale networks we can utilize the explicit expression of the underlying feature map as shown in [25] and set 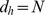. The dual problem corresponding to this primal formulation is given by:
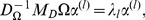
(3)where *M_D_* is the centering matrix which is defined as 
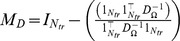
. The 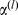 are the dual variables and the kernel function 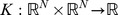 plays the role of similarity function. The dual predictive model is:
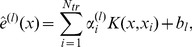
(4)which provides clustering inference for the adjacency list *x* corresponding to the validation/test node *v*.

## Multilevel Hierarchical KSC

We use the predictive KSC model in the dual to get the latent variable matrix for the validation set 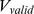 represented as 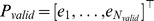 and the test set 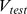 (entire network) denoted by 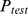. In [27] the authors create an affinity matrix 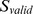 using the latent variable matrix 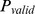 which is a 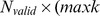-1) matrix, as:
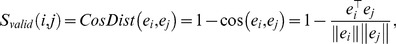
(5)where 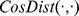 function calculates the cosine distance between 2 vectors and takes values between [0,2]. Nodes which belong to the same community will have 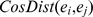 closer to 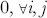 in the same cluster. It was shown in [27] that a rotation of the 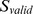 matrix has a block diagonal structure. This block diagonal structure was used to identify the ideal number of clusters *k* in the network using the concept of entropy and balanced clusters.

### Determining the Distance Thresholds

We propose an iterative bottom-up approach on the validation set to determine the set of distance thresholds 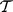. In our approach, we refer to the affinity matrix at the ground level of hierarchy as 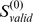. The 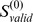 matrix is obtained by calculating the 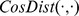 between each element of the latent variable matrix 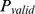 as mentioned earlier. After several empirical evaluations, we observe that distance threshold at level 0 of hierarchy can be set to values between [0.1,0.2]. In our experiments we set 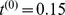. This allows to make the approach tractable to large scale networks which will be explained later.

We then use a greedy approach to select the validation node with maximum number of similar nodes in the latent space i.e we select the projection *e_i_* which has a maximum number of projections *e_j_* satisfying 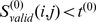. We put the indices of these nodes in 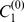 representing the 1*^st^* cluster at level 0 of hierarchy. We then remove these nodes and corresponding entries from 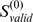 to obtain a reduced matrix. This process is repeated iteratively until 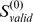 becomes empty. Thus, we obtain the set 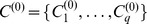 where *q* is the total number of clusters at ground level of hierarchy. The set 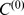 has communities along with the indices of the nodes in these communities.

To obtain the clusters at the next level of hierarchy we treat the communities at the previous levels as nodes. We then calculate the average cosine distance between these nodes using the information present in them. At each level *h* of hierarchy we create a new affinity matrix as:
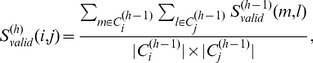
(6)where 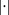 represents the cardinality of the set. In order to determine the threshold at level *h* of hierarchy, we estimate the minimum cosine distance between each individual cluster and the other clusters (not considering itself). Then, we select the mean of these values as the new threshold for that level to combine clusters. This makes the approach different from the classical single-link clustering where we combine two clusters which are closest to each other at a given level of hierarchy and the average-link agglomerative clustering where we combine based on the average distance between all the clusters.

The reason for using mean of these minimum cosine distance values as the new threshold is that if we consider the minimum of all the distance values then there is a risk of only combining 2 clusters at that level. However, it is desirable to combine multiple sets of different clusters. Thus, the new threshold 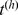 at level *h* is set as:
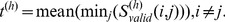
(7)


We use this process iteratively till we reach the coarsest cluster where we have 1 cluster containing all the nodes. As a consequence we obtain the hierarchical clustering 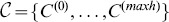 automatically. As we move from one level of hierarchy to another the value of distance threshold increases since we are merging large clusters at coarser levels of hierarchy. We finally end up with a set of increasing distance thresholds 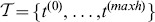.

### Requirements for Feasibility to Large Scale Networks

The whole large scale network is used as test set. The latent variable matrix for the test set is obtained by out-of-sample extensions of the predictive KSC model and defined as 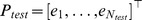. Since we use the entire network as test set, therefore, 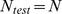. The 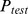 matrix is a 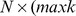-1) dimensional matrix. So, we can store this 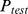 matrix in memory but cannot create an affinity matrix of size 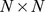 due to memory constraints.

To make the approach feasible to large scale network we put a condition that the maximum size of a cluster at ground level cannot exceed 10,000 (depending on the available computer memory) and the maximum number of clusters allowed at the ground level is 10,000. This limits the size of the affinity matrix at that level of hierarchy to be less than 10,000×10,000. It also effects the choice of the initial value of the distance threshold 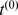. If we set 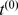 too high (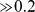) then majority of the nodes at the ground level in the test case will fall in one community resulting in one giant connected component. If we set the value of 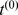 too low (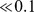) then we will end up with lot of singleton clusters at the ground level in the test case. In our experiments, we observed that the interval any value between [0.1,0.2] is good choice for the initial threshold value at level 0 of hierarchy. To be consistent we chose 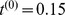 for all the networks.

### Multilevel Hierarchical KSC for Test Nodes

The validation set is a representative subset of the whole network as shown in [26]. Thus, the threshold set 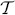 can be used to obtain a hierarchical clustering for the entire network. To make the proposed approach self-tuned, we use 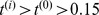, *i*>0, during the test phase.

**Figure 3:**
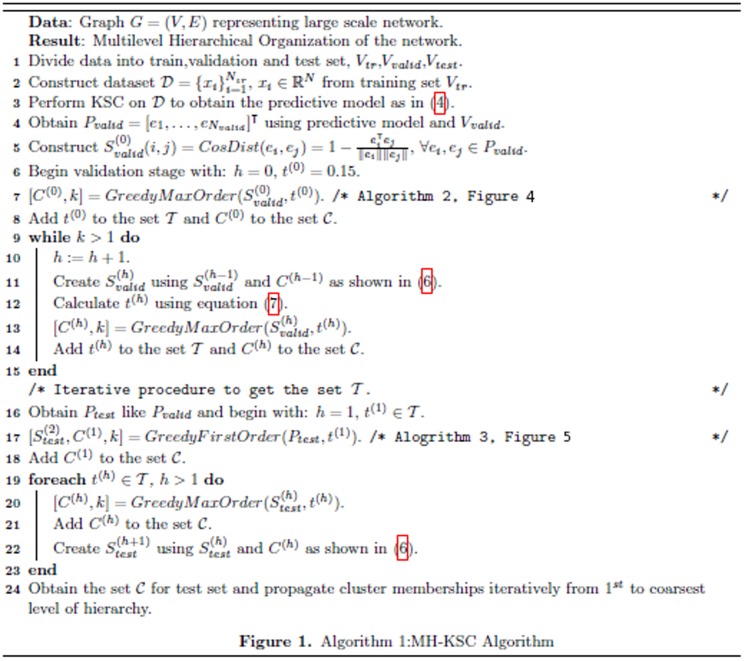
Algorithm 1: MH-KSC Algorithm.

**Figure 4:**
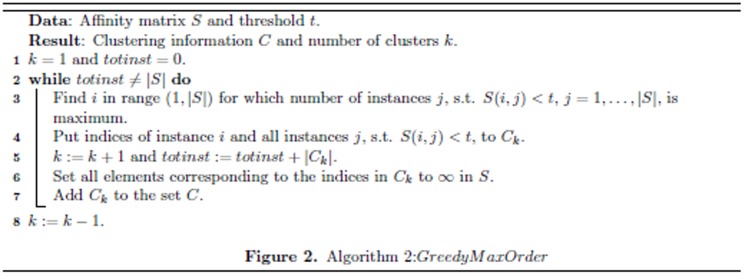
Algorithm 2: *GreedyMaxOrder*.

**Figure 5:**
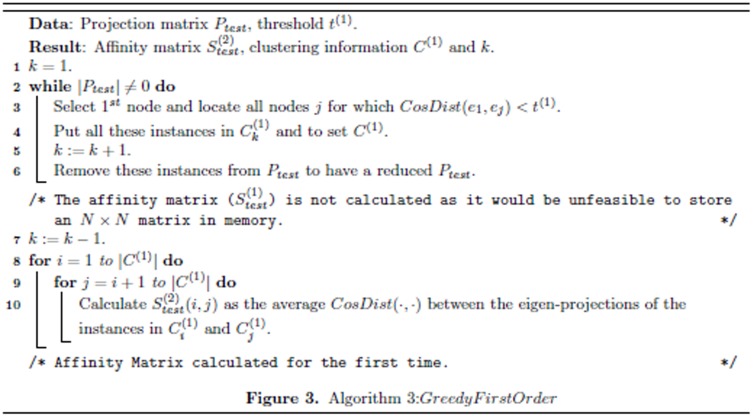
Algorithm 3: *GreedyFirstOrder*.

In order to prevent creating the affinity matrix for the large network we follow a greedy procedure. We select the projection of the first test node and calculate its similarity with the projections of all the test nodes. We then locate the indices (*j*) of those projections s.t. 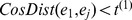. If the total number of such indices is less than 10,000 then we put them in cluster 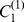 otherwise we select the first 10,000 indices and place them in cluster 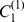. This is due to the constraint that the size of a cluster (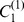) at ground level cannot exceed 10,000. We then remove entries corresponding to those projections in 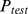 to obtain a reduced matrix. We perform this procedure iteratively until 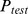 is empty to obtain 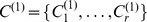 where *r* is the total number of clusters at hierarchical level 1. After the 1*^st^* level, we use the same procedure that was for validation set i.e. creating an affinity matrix at each level using the cluster information along with the threshold set 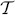 to obtain the hierarchical structure in an agglomerative fashion. The cluster memberships are propagated iteratively from the 1*^st^* level to the highest level of hierarchy. The multilevel hierarchical kernel spectral clustering (MH-KSC) method is described in Figure 3 which refers to Algorithm 2 and Algorithm 3 in Figure 4 and Figure 5 respectively.

### Time Complexity Analysis

The two steps in our proposed approach which require the maximum computation time are the out-of-sample extensions for the test set and the creation of the affinity matrix from the ground level clusters.

Since we use the entire network as test set the time required for out-of-sample extension is 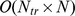. Our greedy procedure to obtain the clustering information at the ground level 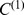 requires 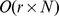 computations where *r* is the number of clusters at 1*^st^* level of hierarchy for the test set. This is because for each cluster 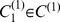 we remove all the indices belonging in that cluster from the matrix 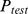. As a result the size of 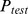 decreases till it reduces to zero resulting in 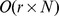 computations. The affinity matrix 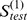 is a symmetric matrix so we only need to compute the upper or the lower triangular matrix. The number of cluster-cluster similarities that we have to calculate is 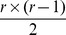 where the size of each cluster at ground level can be maximum 10,000.

However, as shown in [25], we can perform the out-of-sample extensions in parallel on *n* computers and rows of the affinity matrix can also be calculated in parallel thereby reducing the complexity by 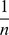.

## Experimental Results

We conducted experiments on 2 synthetic datasets obtained from the toolkit in [4] and 7 real-world networks obtained from Stanford SNAP library (http://snap.stanford.edu/data/index.html).

### Synthetic Network Experiments

The synthetic networks are referred as 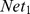 and 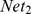 and have 2,000 and 50,000 nodes respectively. The ground truth for these 2 benchmark networks are known at 2 levels of hierarchy. These 2 levels of hierarchy for these benchmark networks are obtained by using 2 different mixing parameters i.e. *μ*_1_ and *μ*_2_ for macro and micro communities. We fixed 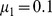 and 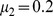 in our experiments. Since the ground truth is known beforehand, we evaluate the communities obtained by our proposed MH-KSC approach using an external quality metric like Adjusted Rand Index (*ARI*) and Variation of Information (*VI*) [33]. We also evaluate the cluster information using internal cluster quality metrics like Modualrity (*Q*) [3] and Cut-Conductance (*CC*) [29]. We compare MH-KSC with Louvain, Infomap and OSLOM.

**Figure 6:**
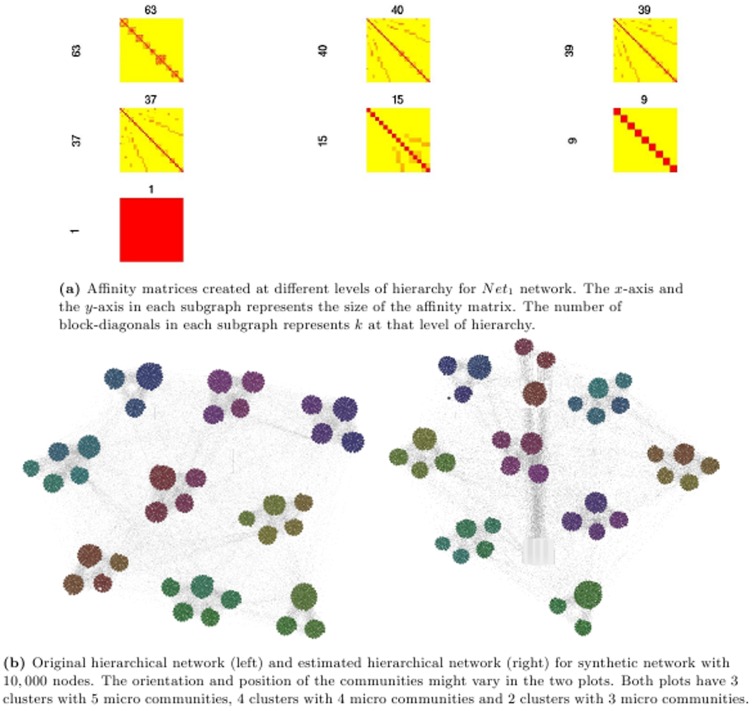
Result of MH-KSC algorithm on benchmark *Net*_1_ network.

**Figure 7:**
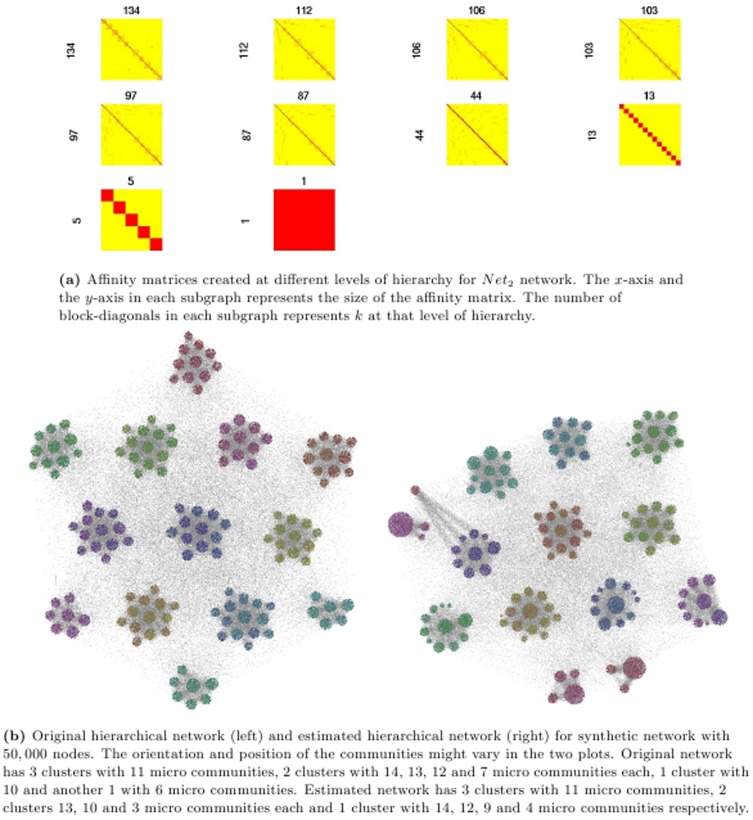
Result of MH-KSC algorithm on benchmark *Net*_2_ network.

Figures 6 and 7 showcase the result of MH-KSC algorithm on the 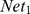 and 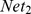 respectively. From Figures 6a and 7a, we observe the affinity matrices generated corresponding to the test set for 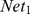 and 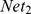 respectively. From Figures 6b and 7b, we can observe the communities prevalent in the original network and the communities estimated by MH-KSC method for 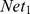 and 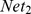 respectively. In 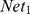 there are 9 macro communities and 37 micro communities while in 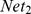 there are 13 macro communities and 141 micro communities as depicted by Figures 6b and 7b.

Table 1 illustrates the first 10 levels of hierarchy for 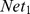 and 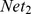 and evaluates the clusters obtained at each level of hierarchy w.r.t. quality metrics *ARI*, *VI*, *Q* and *CC*. Higher values of *ARI* (close to 1) and lower values of *VI* (close to 0) represent good quality clusters. Both these external quality metrics are normalized as shown in [33]. Higher values of modularity (*Q* close to 1) and lower values of cut-conductance (*CC* close to 0) indicate better clustering information.

**Table 1:** Number of clusters (*k*) for top 10 levels of hierarchy by MH-KSC method.

	*Net*_1_					*Net*_2_				
Hierarchy	*k*	*ARI*	*VI*	*Q*	*CC*	*k*	*ARI*	*VI*	*Q*	*CC*
1	-	-	-	-	-	**134**	**0.685**	**0.612**	0.66	**1.98e-05**
2	-	-	-	-	-	112	0.625	0.643	0.685	1.99e-05
3	-	-	-	-	-	106	0.61	0.667	0.691	1.99e-05
4	63	0.972	0.11	0.62	**4.74e-04**	103	0.595	0.692	0.694	**1.98e-05**
5	40	0.996	0.018	0.668	4.86e-04	97	0.53	0.77	0.706	1.99e-05
6	39	**0.996**	**0.016**	0.669	**4.834e-04**	87	0.47	0.90	0.722	1.99e-05
7	**37**	0.965	0.056	0.675	4.856e-04	44	0.636	0.74	**0.773**	1.99e-05
8	15	0.878	0.324	**0.765**	5.021e-04	**13**	**1.0**	**0.0**	**0.82**	2.0e-05
9	**9**	**1.0**	**0.0**	**0.786**	5.01e-04	5	0.12	1.643	0.376	2.12e-05
10	1	0.0	2.19	0.0	5.0e-04	1	0.0	2.544	0.0	2.0e-05

The number of clusters close to the actual number, the best and second best results are highlighted. For 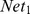 only 7 levels of hierarchy are identified by MH-KSC, rest are represented by ‘-’. The MH-KSC method provides more insight by identifying several meaningful levels of hierarchy with good clusters w.r.t. quality metrics like *ARI*, *VI*, *Q* and *CC*.

Table 2 provides the result of Louvain, Infomap and OSLOM methods and compares it with the best levels of hierarchy for 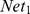 and 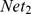. The Louvain, Infomap and OSLOM methods require multiple runs as in each iteration they result in a different partition. We perform 10 runs and report the mean results in Table 2. From Table 2, it can be observed that the best results for Louvain and Infomap methods generally occur at finer levels of hierarchy w.r.t. to *ARI*, *VI* and *Q* metric. Thus, these two methods work well to identify macro communities. The Louvain method works the better than MH-KSC for 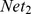 at macro and micro level. However, it cannot obtain similar quality micro communities when compared with MH-KSC method for 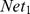 as inferred from Table 2. The Infomap method performs the worst among all the methods w.r.t. detection of communities at coarser levels of granularity. OSLOM performs well w.r.t. to locating both macro communities for 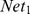 and micro communities for 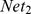 as observed from Table 2. It performs better than any method w.r.t. locating micro communities for 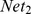 w.r.t. *ARI* and *VI* metric. However, it performs worst while trying to identify the macro communities for the same benchmark network. The MH-KSC performs best on 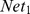 while it performs better w.r.t. locating macro communities for 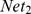.

**Table 2:** 2 best level of hierarchy obtained by Louvain, Infomap, OSLOM and MH-KSC methods on 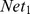 and 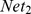 benchmark networks.

Method	*Net*_1_						*Net*_2_					
	Level	k	*ARI*	*VI*	*Q*	*CC*	Level	k	*ARI*	*VI*	*Q*	*CC*
**Louvain**	1	32	0.84	0.215	**0.693**	4.87e-05	1	135	0.853	0.396	**0.687**	**1.98e-05**
	2	9	**1.0**	**0.0**	**0.786**	**5.01e-04**	3	13	**1.0**	**0.0**	**0.82**	**2.0e-05**
**Infomap**	1	8	0.915	0.132	0.771	5.03e-04	1	590	0.003	8.58	0.003	**1.98e-05**
	2	6	0.192	1.965	0.487	5.07e-04	3	13	**1.0**	**0.0**	**0.82**	**2.0e-05**
**OSLOM**	1	38	0.988	0.037	0.655	4.839e-04	1	141	**0.96**	**0.214**	0.64	2.07e-05
	2	9	**1.0**	**0.0**	**0.786**	**5.01e-04**	2	29	0.74	0.633	076	2.08e-05
**MH-KSC**	2	39	**0.996**	**0.016**	0.67	**4.83e-04**	3	134	0.685	0.612	0.66	**1.98e-05**
	5	9	**1.0**	**0.0**	**0.786**	**5.01e-04**	10	13	**1.0**	**0.0**	**0.82**	**2.0e-05**

The best results w.r.t. various quality metrics when compared with the ground truth communities for each benchmark network is highlighted.

### Real-Life Network Experiments

We experimented on 7 real-life networks from the Stanford SNAP datasets. These networks are anonymous networks and are converted to undirected and unweighted networks before performing experiments on them. Table 3 provides information about topological characteristics of these real-life networks. The Fb and Epn networks are social networks, PGP is a trust based network, Cond is a collaboration network between researchers, Enr is an email network, Imdb is an actor-actor collaboration network and Utube is a web graph depicting friendship between the users of Youtube.

**Table 3:** Nodes (V), Edges (E) and Clustering Coefficients (CCF) for each network.

Network	Nodes	Edges	CCF
**Facebook (Fb)**	4,039	88,234	0.6055
**PGPnet (PGP)**	10,876	39,994	0.008
**Cond-mat (Cond)**	23,133	186,936	0.6334
**Enron (Enr)**	36,692	367,662	0.497
**Epinions (Epn)**	75,879	508,837	0.1378
**Imdb-Actor (Imdb)**	383,640	1,342,595	0.453
**Youtube (Utube)**	1,134,890	2,987,624	0.081

In case of real-life networks the true hierarchical structure is not known beforehand. Hence, it is important to show whether they exhibit hierarchical organization which can be tested by identifying good quality clusters w.r.t. internal quality metrics like *Q* and *CC* at multiple levels of hierarchy.

We showcase the results for 10 levels of hierarchy in a bottom-up fashion for the MH-KSC method in Table 4. The finest level of hierarchy has all nodes in one community and is not very insightful. Clusters at finer levels of granularity comprises giant connected components. So, it is more meaningful to give more emphasis to fine grained clusters at coarser levels of hierarchy. To show that real-life networks exhibit hierarchy we evaluate our proposed MH-KSC approach in Table 4.

**Table 4:** Results on MH-KSC algorithm on 7 real-life networks using quality metrics *Q* and *CC*.

		Hierarchical Organization									
Network	Metrics	Level 1	Level 2	Level 3	Level 4	Level 5	Level 6	Level 7	Level 8	Level 9	Level 10
	*k*	358	192	152	121	105	90	71	43	37	21
Fb	*Q*	0.604	0.764	0.769	0.789	0.792	0.81	0.812	0.818	0.821	**0.83**
	*CC*	**2.47e-05**	1.56e-04	2.38e-04	1.91e-04	1.95e-04	1.63e-04	2.16e-04	1.76e-04	2.44e-04	2.4e-04
	*k*	345	274	202	156	129	83	59	46	24	19
PGP	*Q*	0.682	0.693	0.705	0.715	0.725	0.727	0.728	**0.729**	0.701	0.698
	*CC*	8.48e-05	9.84e-05	5.88e-05	1.38e-04	7.2e-05	8.03e-05	1.0e-04	1.07e-04	4.13e-04	**4.89e-05**
	*k*	2676	1171	621	324	171	102	80	58	41	24
Cond	*Q*	0.5	0.567	0.586	0.611	**0.615**	0.614	0.582	0.582	0.574	0.515
	*CC*	2.49e-05	2.6e-05	3.7e-05	3.52e-05	3.6e-05	5.86e-05	2.37e-05	3.45e-05	1.43e-05	**1.4e-05**
	*k*	2208	1002	464	303	211	163	119	76	59	48
Enr	*Q*	0.30	0.388	0.444	0.451	**0.454**	0.427	0.43	0.325	0.328	0.271
	*CC*	**1.19e-05**	3.18e-05	3.1e-05	5.3e-05	7.04e-05	2.69e-04	2.2e-03	1.651e-04	2.56e-05	5.46e-05
	*k*	8808	3133	1964	957	351	220	166	97	66	26
Epn	*Q*	0.105	0.156	0.158	0.176	0.184	0.183	**0.186**	0.184	0.146	0.006
	*CC*	**1.4e-06**	3.1e-06	6.4e-06	7.0e-06	9.5e-06	1.26e-05	7.0e-06	9.0e-06	2.42e-05	7.8e-06
	*k*	7431	1609	890	468	313	200	130	72	46	21
Imdb	*Q*	0.357	0.47	0.473	0.485	0.503	**0.521**	0.508	0.514	0.513	0.406
	*CC*	1.43e-06	2.78e-06	2.79e-06	5.6e-06	4.24e-06	5.6e-06	6.42e-06	1.99e-06	7.46e-06	**9.2e-07**
	*k*	9984	2185	529	274	180	131	100	71	46	26
Utube	*Q*	0.524	0.439	0.679	**0.682**	0.599	0.491	0.486	0.483	0.306	0.303
	*CC*	**2.65e-07**	3.0e-07	1.3e-06	2.4e-06	1.0e-06	7.6e-06	1.03e-5	1.07e-05	2.33e-05	1.55e-04

The best results corresponding to each metric for individual networks are highlighted.

We compare MH-KSC algorithm with Louvain [15], Infomap [7] and OSLOM [21]. We perform 10 runs for each of these methods as they generate a separate partition each time when they are executed. The mean results of Louvain method is reported in Table 5. Table 6 showcases the results for Infomap and OSLOM method.

**Table 5:** Results of Louvain method on 7 real-life networks indicating the top 6 levels of hierarchy.

		Hierarchical Organization					
Network	Metrics	Level 1	Level 2	Level 3	Level 4	Level 5	Level 6
	*k*	-	-	-	225	155	151
Fb	*Q*	-	-	-	0.82	0.846	**0.847**
	*CC*	-	-	-	**9.88e-05**	1.33e-04	1.32e-04
	*k*	-	-	2392	566	154	100
PGP	*Q*	-	-	0.705	0.857	0.882	**0.884**
	*CC*	-	-	**4.95e-05**	8.66e-05	6.8e-05	1.0e-04
	*k*	-	-	6732	1825	1066	1011
Cond	*Q*	-	-	0.56	0.7	0.731	**0.732**
	*CC*	-	-	**1.56e-05**	2.97e-05	3.49e-05	4.15e-05
	*k*	-	-	4001	1433	1237	1230
Enr	*Q*	-	-	0.546	0.608	0.613	**0.614**
	*CC*	-	-	**1.28e-05**	1.88e-05	4.58e-05	6.48e-05
	*k*	10351	2818	1574	1325	1301	1300
Epn	*Q*	0.287	0.319	0.323	0.324	0.324	**0.324**
	*CC*	**1.86e-06**	4.2e-06	4.25e-06	5.57e-06	6.75e-06	1.13e-05
	*k*	-	22613	4544	3910	3815	3804
Imdb	*Q*	-	0.591	0.727	0.729	0.729	**0.729**
	*CC*	-	**1.0e-06**	1.0e-06	1.85e-06	2.5e-06	2.82e-06
	*k*	33623	11587	6964	6450	6369	6364
Utube	*Q*	0.696	0.711	0.714	0.715	0.715	**0.715**
	*CC*	**1.38e-06**	2.22e-06	3.25e-06	3.98e-06	4.06e-06	9.96e-06

The best results are highlighted and ‘-’ is used in of absence of available partitions.

**Table 6:** Results of Infomap and OSLOM methods.

		Infomap		OSLOM				
		Hierarchical Info		Hierarchical Info				
Network	Metrics	Level 1	Level 2	Level 1	Level 2	Level 3	Level 4	Level 5
	*k*	325	131	-	161	50	27	21
Fb	*Q*	0.055	**0.763**	-	0.045	0.133	0.352	**0.415**
	*CC*	**2.86e-05**	2.3e-04	-	**2.0e-04**	2.0e-04	3.0e-04	3.0e-04
	*k*	85	65	431	143	51	48	45
PGP	*Q*	0.041	**0.862**	0.748	**0.799**	0.709	0.709	0.709
	*CC*	1.66e-04	**1.40e-04**	1.74e-04	**5.32e-05**	2.06e-04	1.56e-04	6.64e-05
	*k*	1009	173	4092	2211	1745	1613	1468
Cond	*Q*	**0.648**	0.027	0.483	0.574	0.615	**0.615**	0.05
	*CC*	**1.71e-05**	2.78e-05	1.77e-05	2.48e-05	3.04e-05	6.56e-05	**1.16e-05**
	*k*	1920	1084	-	3149	2177	2014	1970
Enr	*Q*	0.015	**0.151**	-	0.317	0.382	0.412	**0.442**
	*CC*	**1.83e-05**	8.39e-04	-	**1.75e-05**	4.96e-05	9.92e-05	7.22e-05
	*k*	14170	50	1693	584	206	30	25
Epn	*Q*	5.3e-06	**4.48e-04**	0.162	0.226	**0.239**	0.098	0.019
	*CC*	**3.97e-06**	4.63e-05	1.23e-05	9.75e-06	2.45e-05	8.2e-06	**7.9e-06**
	*k*	14308	3238	-	7469	2639	2017	2082
Imdb	*Q*	0.04	**0.707**	-	0.045	0.092	0.1	0.115
	*CC*	**1.23e-06**	4.72e-06	-	**1.35e-06**	2.03e-06	7.95r-06	1.17e-05
	*k*	10703	976	18539	6547	4184	2003	1908
Utube	*Q*	0.035	**0.698**	0.396	0.53	**0.588**	0.487	0.027
	*CC*	**1.38e-06**	5.56e-06	1.52e-06	3.1e-07	**2.72e-07**	6.1e-06	5.69e-06

The best results for each method corresponding to each network is highlighted and ‘-’ represent not applicable cases.

From Table 5 it is evident that the Louvain method works best w.r.t. the modularity (*Q*) criterion. This aligns with methodology as it is trying to optimize for *Q*. However, the Louvain method always performs worse than MH-KSC algorithm w.r.t. cut-conductance *CC* as observed from Tables 4 and 5. Another issue with the Louvain method is that except for the Fb and PGP networks it is not able to detect (<1000 clusters) high quality clusters at finer levels of granularity. This is attributed to the resolution limit problem suffered by Louvain method. From Table 6 we observe that the Infomap method produces only 2 levels of hierarchy. In most of the cases, the clusters at one level of hierarchy perform good w.r.t. only 1 quality metric except the PGP and Cond networks. The difference between the quality of the clusters at the 2 levels of hierarchy is quite drastic. This reflects that the Infomap method is not very consistent w.r.t. various quality metrics.

We compare the performance of MH-KSC method with OSLOM in detail. From Tables 4 and 5 we observe that the MH-KSC technique outperforms OSLOM w.r.t. both quality metrics for Fb, Enr, Imdb and Utube networks while OSLOM does the same only for Cond network. In case of PGP, Cond and Epn networks OSLOM results in better *Q* than MH-KSC. However, MH-KSC approach has better *CC* value for PGP and Epn networks. For large scale networks like Enr, Imdb and Utube, OSLOM cannot identify good quality coarser clusters i.e. number of clusters detected are always >1000.

### Visualization and Illustrations

**Figure 8:**
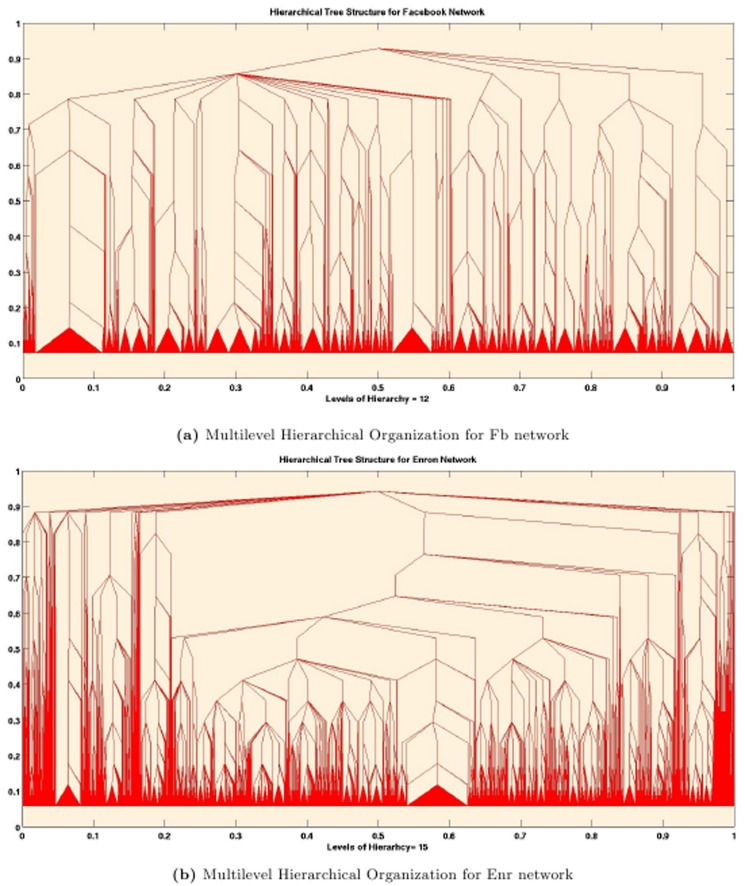
Tree based visualization of the multilevel hierarchical organization prevalent in 2 real-life networks.

We provide a tree based visualization of the multilevel hierarchical organization for Fb and Enr networks in Figure 8. The hierarchical structure is depicted as tree for Fb and Enr network in Figures 8a and 8b respectively.

**Figure 9:**
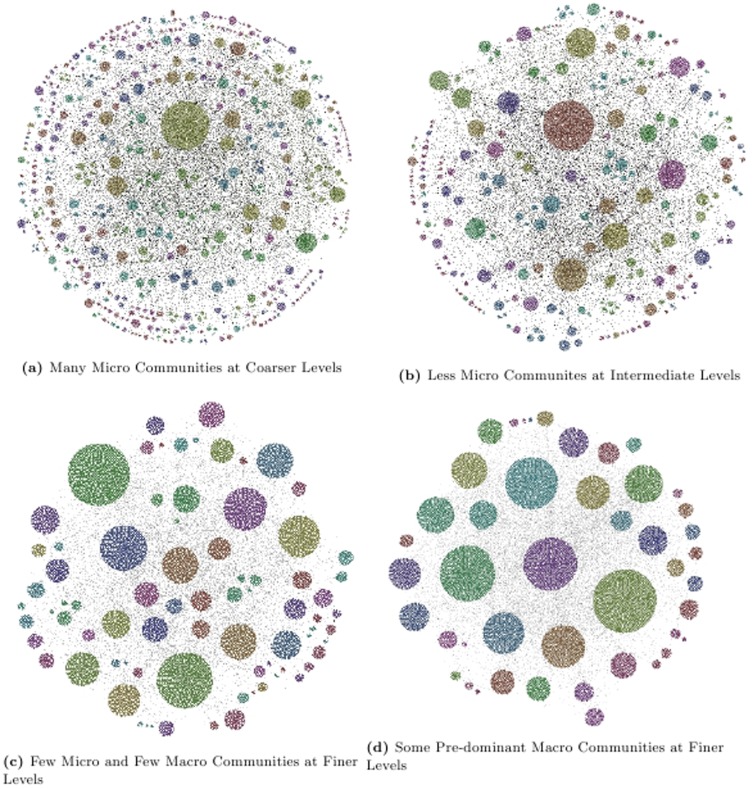
MH-KSC algorithm for the PGP network. Communities with same colour belong to one cluster.

**Figure 10:**
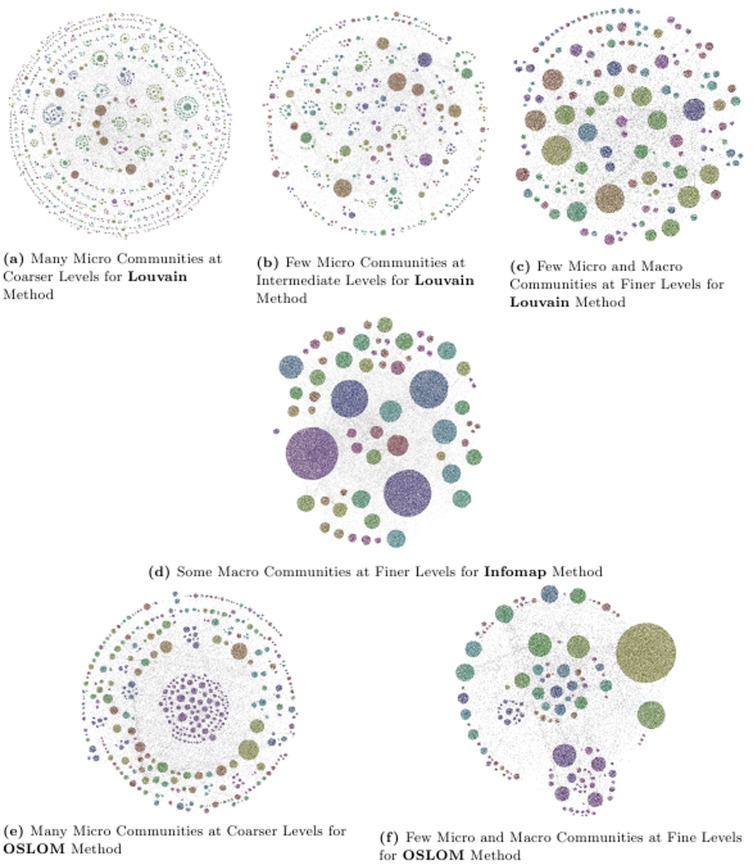
Results of Louvain, Infomap and OSLOM methods for PGP network.

We plot the results corresponding to fine, intermediate and coarse levels of hierarchy for PGP network using the software provided in [21]. The software requires all the nodes in the network along with 2 levels of hierarchy. In Figure 9 we plot the results for PGP net corresponding to MH-KSC algorithm using 2 fine, 4 intermediate and 2 coarse levels of the hierarchical organization. For Louvain method we use 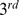 and 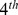 level of hierarchy as inputs for the fine clusters, 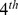 and 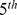 level of hierarchy as inputs for intermediate clusters and 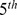 and 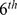 level of hierarchy as inputs for plotting coarsest clusters. The Infomap method only generates 2 level of hierarchy which correspond to a plot for coarse clusters. Similarly, for OSLOM we plot coarse and fine clusters. The results for Louvain, Infomap and OSLOM methods are depicted in Figure 10.

Figures 9 and 10 show that MH-KSC algorithm allows to depict richer structures than the other methods. It has more flexibility and allows the visualization at coarser, intermediate and finer levels of granularity. From Figures 10a, 10b, 10c and Table 5, we observe that the Louvain method can only detect quality clusters at coarser levels of granularity and cannot detect less than 1,00 communities. While the Infomap method can only locate giant connected components for the PGP network as observed from Figure 10d and Table 6. The OSLOM method also seems to work reasonably well as observed from Figures 10e and 10f. However, it detects fewer levels of hierarchy and thus has less flexibility in terms of selection for the level of hierarchy than the proposed MH-KSC approach.

**Figure 11:**
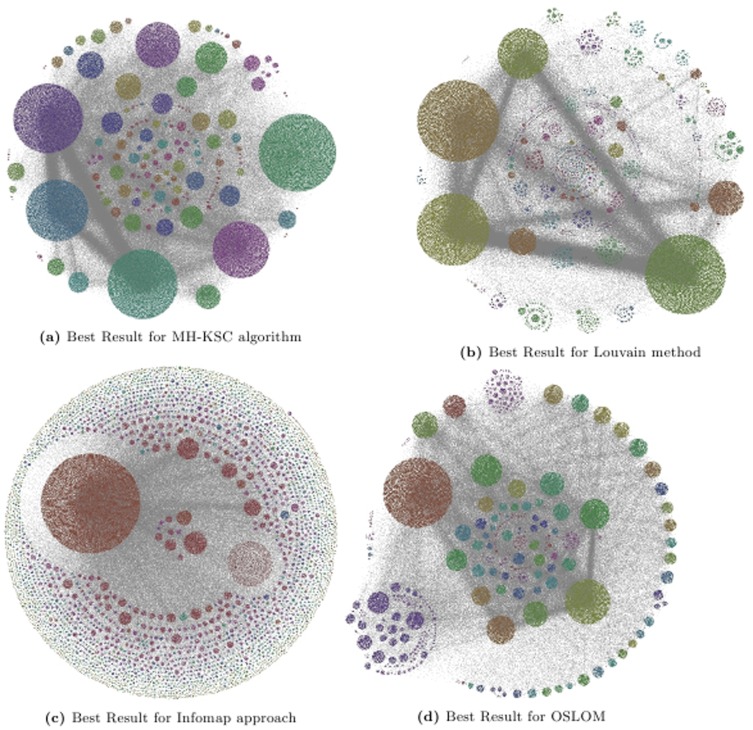
Representing the 2 best levels of hierarchy for Epn network w.r.t. modularity criterion.

**Figure 12:**
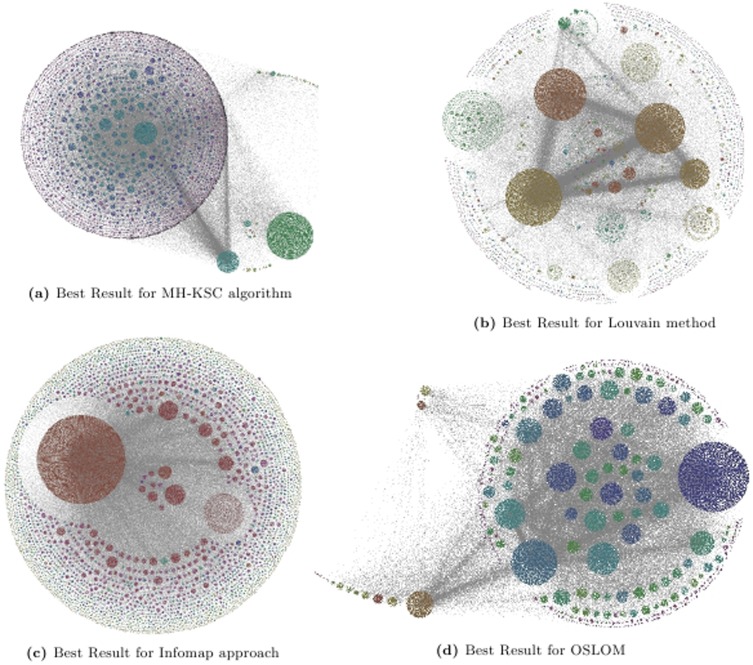
Representing the 2 best levels of hierarchy for Epn network w.r.t. cut-conductance criterion.

We provide a visualization of the 2 best layers of hierarchy for Epn network based on the *Q* and the *CC* criterion for MH-KSC, Louvain, Infomap and OSLOM methods respectively in Figures 11 and 12. The result for Infomap method in both the figures is the same as it only generates 2 levels of hierarchy.

## Conclusions

We proposed a new multilevel hierarchical kernel spectral clustering (MH-KSC) algorithm. The approach relies on the KSC primal-dual formulation and exploits the structure of the projections in the eigenspace. The projections of the validation set provided a set (*T*) of increasing distance thresholds. These distance thresholds were used along with affinity matrix obtained from the projections in an iterative procedure to obtain a multilevel hierarchical organization in a bottom-up fashion. We highlighted some of the necessary conditions for the feasibility of the approach to large scale networks. We showed that many real-life networks exhibit hierarchical structure. Our proposed approach was able to identify good quality clusters for both coarse as well as fine levels of granularity. We compared and evaluated our MH-KSC approach against several state-of-the-art large scale hierarchical community detection techniques.
